# Quadricuspid Aortic Valve with Ruptured Sinus of Valsalva Aneurysm: a Case Report

**DOI:** 10.21470/1678-9741-2020-0735

**Published:** 2023

**Authors:** Shuran Huang, Xiaolong Liu, Zhanguo Sun

**Affiliations:** 1 Department of Intensive Care Unit, Affiliated Hospital of Jining Medical University, Shandong, People’s Republic of China.; 2 Department of Medical Imaging, Affiliated Hospital of Jining Medical University, Shandong, People’s Republic of China.

**Keywords:** Quadricuspid Aortic Valve, Sinus of Valsalva, Aortic Aneurysm, Computed Tomography Angiography, Echocardiography, Heart Atria

## Abstract

Quadricuspid aortic valve (QAV) and sinus of Valsalva aneurysm (SVA) are rare congenital anomalies. We report an elderly patient with QAV associated with a ruptured SVA to the right atrium. Transthoracic echocardiographic and computed tomographic images are presented. We emphasize the important role of computed tomography angiography in establishing and confirming the diagnosis and facilitating treatment planning. The patient was successfully operated by a minimally invasive approach.

## INTRODUCTION

The most common type of aortic valve deformation is the bicuspid valve, followed by the unicuspid valve. Quadricuspid aortic valve (QAV) is a rare entity^[1]^. Sinus of Valsalva aneurysm (SVA) is also an uncommon disorder which is usually caused by congenital weakness of the sinus wall. SVA is typically associated with a bicuspid aortic valve. Only few cases of the unusual association between QAV and SVA have been reported till date. We present an elderly patient who had QAV associated with a ruptured SVA to the right atrium. The diagnosis was established by transthoracic echocardiography (TTE) and computed tomography angiography (CTA). We highlight the important role of CTA in the preoperative workup of these patients. The patient was successfully operated via a minimally invasive approach.

## CASE REPORT

**Fig. 1:**
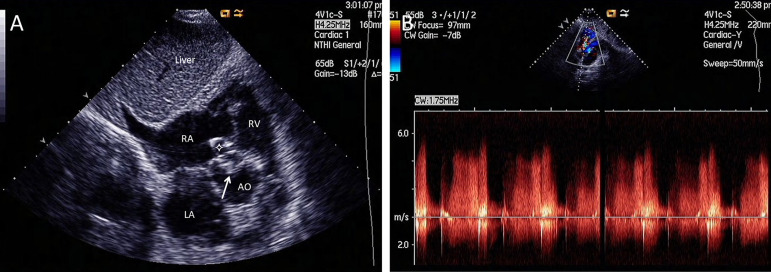
A) Subcostal short axis view of transthoracic echocardiogram showing thickened sinus of the accessory aortic cusp (arrow) with a windsock (four-point star) deformity and a shunt from right-aortic cusp to right atrium during systole and diastole by color Doppler. B) Continuous wave Doppler showing the shunt from left to right, most evident during diastole and attenuated during systole; the velocity of the shunt was 5.7 m/s. AO=aortic root; LA=left atrium; RA=right atrium; RV=right ventricle

A 63-year-old Chinese man presented with a history of shortness of breath and chest distress for seven months. His symptoms had aggravated in the last 10 days. His past medical history was unremarkable. Physical examination revealed a 3/6 diastolic murmur at 2-4 intercostal spaces along the left sternal border. Chest X-ray showed severe pulmonary emphysema. Electrocardiography (ECG) findings were normal. Because of pulmonary emphysema, TTE provided only limited findings; we obtained the images from the subcostal window instead of parasternal windows. The size of the left and right ventricles was normal, left ventricular ejection fraction was 61%, and diastolic function of the left ventricle was impaired in stage I. Subcostal short axis view showed thickening of aortic valve leaflets with mild aortic regurgitation; however, the number of aortic cusps could not be determined. Color Doppler revealed windsock deformity with a shunt from the accessory aortic cusp to the right atrium during systole and diastole. The velocity of the shunt was 5.7 m/s (Figure 1). Transesophageal echocardiography (TEE) was refused by the patient.

**Fig. 2:**
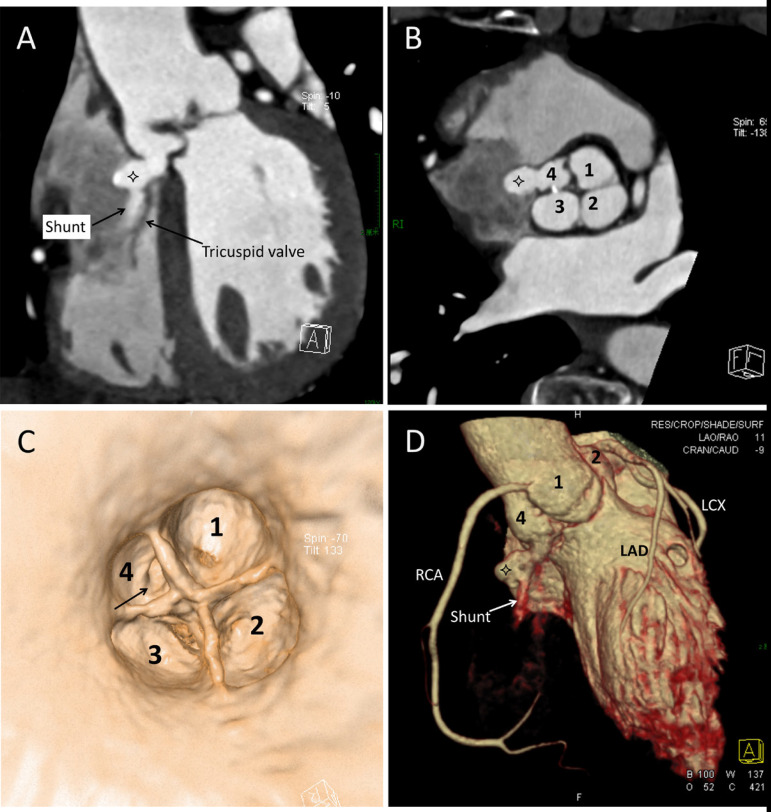
A) The right atrium has lesser amount of contrast material than the aorta, allowing visualization of a jet from the aorta to the right atrium though the windsock (four-point star). B) Multiplanar reconstruction in diastole by computed tomography angiography showing thickened aortic leaflets, especially the accessory one. A windsock (four-point star) developed from the accessory sinus. C) Virtual intra-aortic endoscopy image showing a quadricuspid aortic valve with three equal-sized cusps (1-3) and a smaller accessory cusp (4); the sinus of Valsalva aneurysm originated from the accessory sinus (arrow). D) Volume rendering image showing normal coronary arteries and the windsock (four-point star) with a shunt (arrow). 1=right coronary sinus; 2=left coronary sinus; 3=non-coronary sinus; 4=accessory sinus; LAD=left anterior descending artery; LCX=left circumflex coronary artery; RCA=right coronary artery

As part of chest distress evaluation and to rule out any associated coronary artery disease, an ECG-gated CTA scan was performed using dual-source computed tomography (SOMATOM definition, Siemens, Forchheim, Germany) with ECG-gated tube current modulation (tube voltage: 100 kV; full tube current was applied from 30% to 75% of the R-R interval). It revealed a QAV with three equal-sized cusps and a smaller accessory cusp (Figure 2). All the aortic valve leaflets were thickened, especially the accessory one, which was located between the right coronary sinus and the non-coronary sinus with mild calcification at its irregular margin. Different types of reconstruction, including multiplanar reconstruction, volume rendering, and virtual endoscopy, indicated a windsock fistulous connection between the accessory cusp and the right atrium, which projected along and just above the septal leaflet of the tricuspid valve. The windsock was 14 mm in length and 7 mm in width, with a 5-mm crevasse underneath. There was a diastolic shunting through the windsock into the right atrium, which appeared as a jet of blood with greater contrast material extending from the aorta into the right atrium (Figure 2). Nevertheless, the images reconstructed in systole demonstrated that the left-to-right shunt was less than in diastole, possibly due to the obstruction of the corresponding opened aortic valve leaflet. Multiphase reconstructed imaging indicated that the flow through the communication was predominately diastolic; however, some flow persisted during systole. Coronary arteries were normal with no plaque or deformity (Figure 2).

**Fig. 3:**
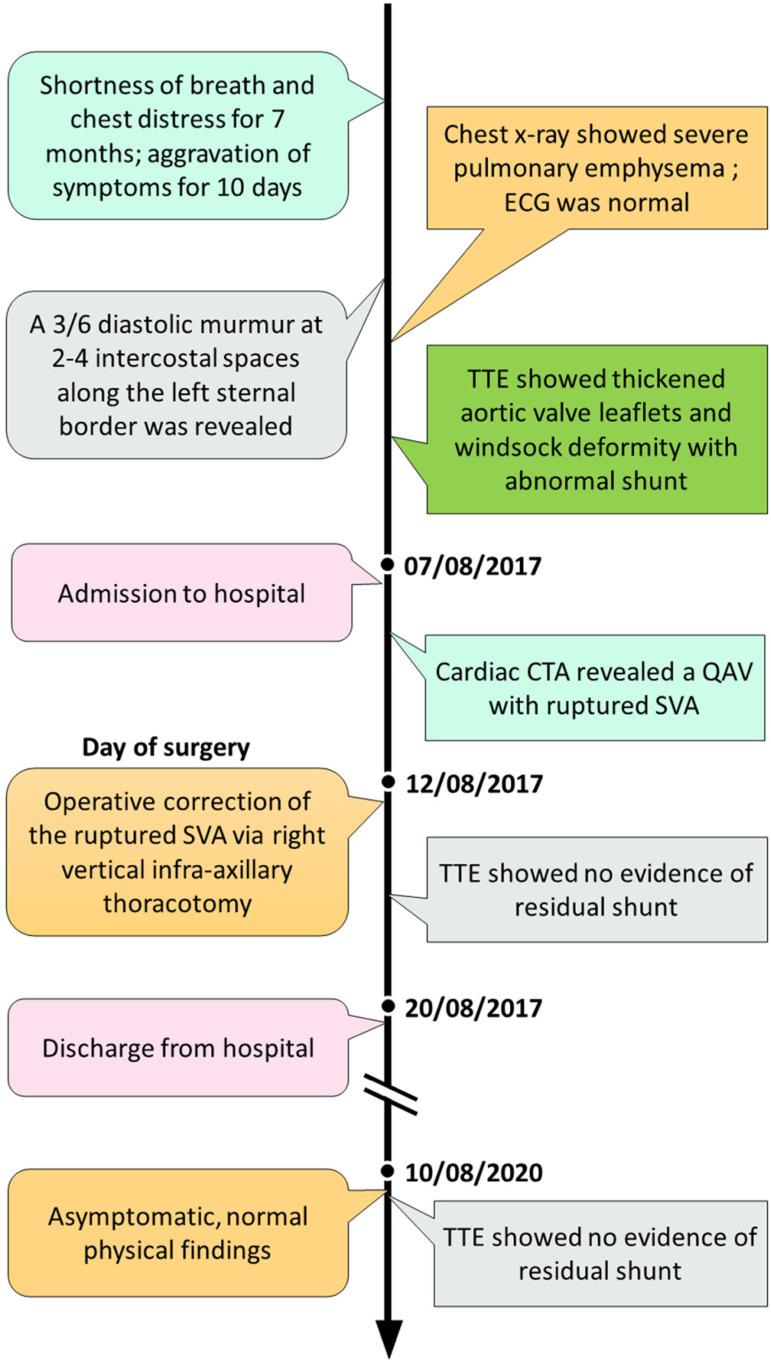
Timeline of the diagnosis and treatment process in the present case. CTA=computed tomography angiography; ECG=electrocardiography; QAV=quadricuspid aortic valve; SVA=sinus of Valsalva aneurysm; TTE=transthoracic echocardiography

The patient had no surgical indication for valve-replacement. Therefore, surgical correction of the ruptured SVA was performed. A small, right vertical infra-axillary thoracotomy was used. After entering the right chest at the fourth intercostal space, the right atrium was opened, and a windsock deformity was observed. The windsock in the right atrium was excised, and the remaining defect was closed by a “figure of eight” suture with 4-0 Prolene® string and reinforced by a Dacron® patch. Postoperative echocardiography showed no signs of residual shunt. The postoperative period was uneventful, and the patient was discharged eight days after operation. At 36-month follow-up, the patient has remained asymptomatic, and echocardiography revealed no progression of aortic regurgitation (Figure 3).

## DISCUSSION

QAV and SVA are rare congenital anomalies. To the best of our knowledge, only four case reports describing the unusual association between QAV and ruptured SVA have been published^[2-5]^. Our patient is the first such documented Chinese patient who was diagnosed by TTE and CTA; the operation was successfully performed by a minimally invasive approach.

A summary of the previously reported cases (including the present case) is presented in Table 1. Age of onset in previous cases indicates that patients with these congenital anomalies typically become symptomatic in the prime of their life. However, some cases may not develop severe clinical manifestations, as seen in our case. The symptoms of all five patients are described in Table 1; four of these patients had a definite time of onset of symptoms, lasting from days to months. The very recent onset of symptoms suggested that the lesions were functionally well tolerated until the SVA ruptured caused it^[4]^.

**Table 1:** Summary of previously reported cases of QAV associated with ruptured SVA (including the present case).

Author	Age (years)	Sex	Symptoms	Type of QAV	AR	Origin of SVA	Ruptured into	Diagnostic method	Operation
Unger P^[2]^	41	Male	Exertional dyspnea and peripheral edema for 8 days	Not described	Mild	Right sinus	RVOT	TTE	Not described
Aggarwal SK^[3]^	32	Male	Sudden onset of chest pain 5 months earlier followed by exertional dyspnea	Type A	Mild to moderate	Right anterior non-coronary sinus	RA	2D and 3D TTE, CC	Correction of SVA
Yang EH^[4]^	22	Female	Gradually worsening dyspnea on exertion, orthopnea, and cough for 2 weeks	Type B	Severe	Right sinus	RA	TTE	Resection of the SVA; aortic valve replacement
Akerem Khan SK^[5]^	24	Female	Intermittent chest pain, palpitations, and progressive dyspnea on exertion for several weeks	Not described	Not described	Right anterior non-coronary sinus	RA	TTE, TEE,	Correction of SVA (via median sternotomy)
								CTA	
Present case	61	Male	Chest distress and shortness of breath for 7 months; symptom aggravation for 10 days	Type B	Mild	Right anterior non-coronary sinus	RA	TTE, CTA	Repaired SVA (via RVAT)

2D=two dimensional; 3D=three dimensional; AR=aortic regurgitation; CC=cardiac catheterization; CTA=computed tomography angiography; QAV=quadricuspid aortic valve; RA=right atrium; RVAT=right vertical infra-axillary thoracotomy; RVOT=right ventricular outflow tract; SVA=sinus of Valsalva aneurysm; TEE=transesophageal echocardiography; TTE=transthoracic echocardiography

According to the size of each individual aortic valve cusp, QAV has been classified into seven different subtypes^[5]^. The two most frequent types are type A (four equal cusps) and type B (three equal cusps with one smaller cusp). Type B QAVs reportedly have a higher proportion of normally functional valves than type A^[4]^. In two of the four previously reported cases, the type of QAV was not clearly described; of the remaining two cases, one patient had type A, while the other patient had type B QAV; our case also had type B QAV. In all five patients described in Table 1, SVA of patients with QAV usually developed from the right anterior non-coronary sinus (3/5) and projected into the right atrium (4/5). Infrequently, SVA may originate in the right coronary sinus (2/5) and project in the outflow tract of the right ventricle (1/5).

Accurate anatomical and functional assessment is an essential prerequisite for minimally invasive treatment of cardiac anomalies. TTE is a universally used technique; however, its use is limited in patients with severe pulmonary emphysema due to the limited echo window. In this case, preoperative TTE images did not allow for adequate anatomical delineation, even though it provided valuable hemodynamic information. TEE is considered as a better imaging modality as it provides a clear anatomical image of aortic valves and coronary ostia and can confirm the diagnosis of QAV as well as SAV^[5]^.

Multidetector CTA with its excellent temporal and spatial resolution and advanced post-processing reconstructions can allow detailed anatomical assessment of the aortic root structure. In our patient, it provided clear images of QAV, ruptured SAV, and the normal coronary arteries. This approach is helpful when the TTE window limits visualization and is an alternative to TEE for preoperative planning.

Among the five cases described in Table 1, the surgical treatment can be classified into two categories. Aortic valve replacement and resection of SVA were done in one patient with severe aortic regurgitation, while simple correction of SVA was performed in four patients with mild to moderate aortic regurgitation. The surgical indication for QAV depends on the severity of aortic regurgitation; however, surgical management of SVA is essential once it is diagnosed.

In our case, right vertical infra-axillary thoracotomy was used for resecting the SVA to avoid the standard median sternotomy and the associated discomfort. This port-access approach is widely used for mitral valvuloplasty, mitral valve replacement, atrial septal defect closure, and repair of other simple congenital heart defects; in addition, it provides excellent cosmetic and clinical outcomes^[6]^. After resection of SVA, close follow-up assessment is required to monitor for progression of aortic regurgitation.

## CONCLUSION

We reported a case of a patient who had QAV in association with ruptured SVA to the right atrium and reviewed the pertinent literature. Patients with this rare congenital anomaly typically show symptoms in early adulthood and present with a very recent onset of symptoms caused by SVA rupture. In the previously reported cases, most SVAs associated with QAV originated in the right or non-coronary sinus. TTE is the most widely used noninvasive diagnostic modality in these patients; however, TEE and CTA afford better anatomical and functional characterization of lesions, facilitating preoperative planning. This case report also highlights the use of a minimally invasive approach via a small, vertical right infra-axillary incision to correct the SVA.

## References

[1] Veronese ET, Brandão CMA, Steffen SP, Pomerantzeff P, Jatene FB (2019). Quadricuspid aortic valve: three cases report and literature
review. Braz J Cardiovasc Surg.

[2] Unger P, Preumont N, Stoupel E (2000). Ruptured sinus of Valsalva aneurysm with right ventricular
obstruction, quadricuspid aortic valve, and ventricular septal
defect. Heart.

[3] Aggarwal SK, Lingan A, Reddy KK, Swamy M, Iyer VR, Srivatsa SS (2009). Quadricuspid aortic valve with ruptured sinus of valsalva
aneurysm to the right atrium. Echocardiography.

[4] Yang EH, Rawal M, Pillutla P, Criley JM (2011). Quadricuspid aortic valve with sinus of Valsalva
rupture. Congenit Heart Dis.

[5] Akerem Khan SK, Tamin SS, Burkhart HM, Araoz PA, Young PM (2013). Quadricuspid aortic valve with ruptured sinus of
Valsalva. Cardiol Young.

[6] Kaneda T, Nishino T, Saga T, Nakamoto S, Ogawa T, Satsu T (2013). Small right vertical infra-axillary incision for minimally
invasive port-access cardiac surgery: a moving window method. Interact Cardiovasc Thorac Surg.

